# Contribution of quantitative changes in individual ionic current systems to the embryonic development of ventricular myocytes: a simulation study

**DOI:** 10.1007/s12576-013-0271-x

**Published:** 2013-06-13

**Authors:** Chikako Okubo, Hitomi I. Sano, Yasuhiro Naito, Masaru Tomita

**Affiliations:** 1 Institute for Advanced Biosciences, Keio University, Fujisawa, Kanagawa 252-0882 Japan; 2 Systems Biology Program, Graduate School of Media and Governance, Keio University, Fujisawa, Kanagawa 252-0882 Japan; 3 Department of Environment and Information Studies, Keio University, Fujisawa, Kanagawa 252-0882 Japan

**Keywords:** Computer simulation, Ion channels, Cardiac ventricular cells, Development, Electrophysiology, Spontaneous activity

## Abstract

Early embryonic rodent ventricular cells exhibit spontaneous action potential (AP), which disappears in later developmental stages. Here, we used 3 mathematical models—the Kyoto, Ten Tusscher–Panfilov, and Luo–Rudy models—to present an overview of the functional landscape of developmental changes in embryonic ventricular cells. We switched the relative current densities of 9 ionic components in the Kyoto model, and 160 of 512 representative combinations were predicted to result in regular spontaneous APs, in which the quantitative changes in Na^+^ current (*I*_Na_) and funny current (*I*_f_) made large contributions to a wide range of basic cycle lengths. In all three models, the increase in inward rectifier current (*I*_K1_) before the disappearance of *I*_f_ was predicted to result in abnormally high intracellular Ca^2+^ concentrations. Thus, we demonstrated that the developmental changes in APs were well represented, as *I*_Na_ increased before the disappearance of *I*_f_, followed by a 10-fold increase in *I*_K1_.

**Electronic supplementary material:**

The online version of this article (doi:10.1007/s12576-013-0271-x) contains supplementary material, which is available to authorized users.

## Introduction

Several hundred types of cells develop from a single genome through accurate spatiotemporal regulation of gene expression. The vertebrate heart is a good example of this phenomenon, as it substantially changes its shape and function at the cellular, tissue, and organ levels throughout a lifetime. In early embryonic development, the heart becomes a functional organ, acting as a pump. The heart develops and gains new functions while continuously pumping blood, and heart abnormalities during the early developmental stages progress to congenital heart malformations; therefore, the developmental program of the heart, including the expression of the genes responsible for various ionic channels, is likely to be tightly regulated. Electrophysiological recordings of various ionic channels and quantification of the genes responsible for the channels have been reported primarily for 4 representative stages: early embryonic (EE), late embryonic (LE), neonatal, and adult. To provide a complete overview of developmental regulation, it is necessary to observe the developmental changes occurring in the heart across these representative stages.

In rodents, spontaneous action potentials (APs) have been reported for the EE stage in developing rodent ventricular myocytes, eventually disappearing in passive contracting cells in the LE stage [1]. The electrophysiological properties of individual ion channels have been investigated in isolated ventricular myocytes at the 4 representative stages by means of patch-clamp methods [2–4]. In addition to cells that exhibit spontaneous APs, embryonic rodent ventricular tissues also contain quiescent cells with no spontaneous APs. In 12-day fetal hearts, for example, 9 of 14 ventricular cells were quiescent and exhibited a resting membrane potential (RMP) of −48.4 ± 1.8 mV [5]; similarly, in 18-day postcoitum (dpc) mice, 6 of 13 isolated ventricular cells were spontaneously active, whereas the remaining 7 cells were quiescent [6]. Moreover, the beating rate of the spontaneous APs ranged from 35 ± 11 beats per minute (bpm) [5] to 232 bpm [7] in 12.5-dpc embryonic rat ventricles, and from 178 ± 12.7 [8] to 124 ± 8.7 bpm [9] in 9.5-dpc embryonic mouse ventricles; the reported beating rates roughly correspond to a basic cycle length (BCL) of approximately 259–2,500 ms. In addition to regular spontaneous APs, irregular spontaneous APs have also been reported in the embryonic ventricular cells of both mice [6] and rats [5]. Previously, we modeled the developmental changes in the APs of cardiac ventricular myocytes [10] by using the Kyoto model [11] and the Luo–Rudy dynamic (LRd) model [12]. The measured APs at developmental stages were reproduced using common sets of these models by varying the relative densities of the ionic currents, pumps, exchangers, and sarcoplasmic reticulum (SR) Ca^2+^ kinetics. In addition, Jonsson et al. [13] combined molecular biology and computer simulations to demonstrate that human embryonic stem cell-derived cardiomyocytes (hESC-CMs) have an immature electrophysiological phenotype, based on insufficient function of inward rectifier K^+^ current (*I*_K1_) channels and a shift in the activation of sodium channels. Thus, computer simulation is a powerful approach for confirming experimental data and providing insights into the possible functional mechanisms involved in cardiac development. Although our simulations well reproduced the disappearance of the spontaneous APs between the EE and LE stages as well as the changes in AP durations during the transition from the LE to the neonatal and adult stages, we have not clarified the contribution of each ionic current system to the reported characteristics of rodent ventricular cells.

In the present study, we examined the functional changes in developing embryonic ventricular cells to identify the pivotal components in the model in order to describe the reported characteristics of embryonic rodent ventricular cells. We switched the relative densities of ionic components that differ between the EE and LE stages, and tested 512 combinations with the Kyoto model, 128 combinations with the Ten Tusscher–Panfilov (TP) human ventricular cell model [14], and 32 combinations with the LRd model. The 160 regular spontaneous APs predicted in the Kyoto model had a wide range of BCL values, all of which were within the reported range of the BCL in 9.5-dpc mice [6, 8, 9] and in 11.5-dpc rats [5, 7]. In all 3 models, the combinations in which *I*_K1_ was increased before the disappearance of *I*_f_ were predicted to result in high [Ca^2+^]_i_, and most of the combinations had quiescent membrane potentials slightly positive to −80 mV, as reported in 12.5-dpc fetal rat ventricular cells [5].

## Methods

Previously, we simulated the APs of rodent ventricular cells at the EE, LE, and neonatal stages by using the Kyoto model—an electrophysiological model of guinea pig ventricular cells [10, 11]. Briefly, quantitative changes in various ionic components were represented as the densities of the components in the developmental stages relative to those in the adult stage. These relative densities were then multiplied by the corresponding conductance (nS/pF) or conversion factors (pA/pF·mM) to demonstrate that developmental changes in the APs can be reproduced using common sets of mathematical equations. We adopted the same procedure in the present study, and reconstructed EE and LE ventricular cell models by using the updated Kyoto model [15].

### Ionic currents, exchangers, and SR Ca^2+^ kinetics

**Fig. 1:**
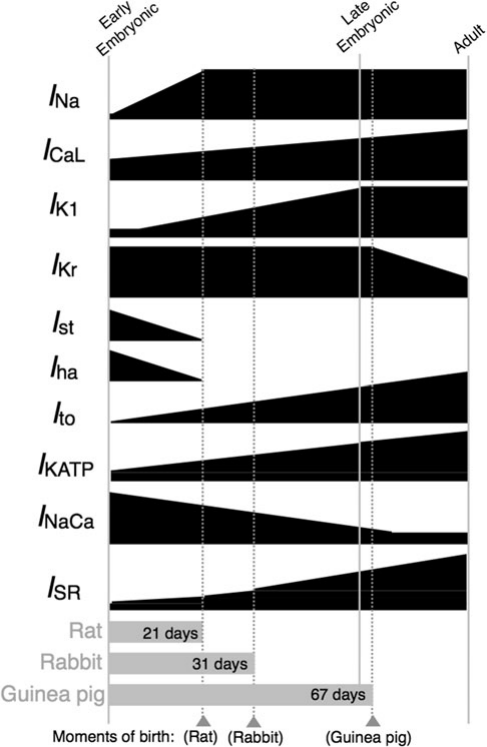
Developmental changes in ionic components of rodent ventricular cells. Relative densities of the Na^+^ current (*I*_Na_), L-type Ca^2+^ current (*I*_CaL_), inward rectifier K^+^ current (*I*_K1_), transient outward current (*I*_to_), and ATP-sensitive K^+^ current (*I*_KATP_) increase during embryonic development (see Table 1 for details). The funny current (*I*_f_) and sustained inward current (*I*_st_) were assumed to completely disappear in late embryonic (LE) ventricular cells (Table 2). The relative densities of the Na^+^/Ca^2+^ exchange current (*I*_NaCa_) and sarcoplasmic reticulum (SR) components are known to change reciprocally [20]; the relative density of *I*_NaCa_ decreases as the SR-related proteins, ryanodine receptor (RyR) proteins and SERCA, increase (Table 3). The *gray bars* indicate the gestational durations of rat [34], rabbit [21], and guinea pig [35]. We defined that the EE stage corresponds to approximately 9.5 dpc in mice and 11.5 dpc in rats, and the LE stage corresponds to 1–5 days before birth

Quantitative changes in ionic currents (Fig. 1) were either computed from current–voltage (*I*–*V*) curves or estimated on the basis of qualitative observations as relative densities. In our previous study [10], we showed that the EE stage corresponds to approximately 9.5 dpc in mice and 11.5 dpc in rats, and the LE stage corresponds to 1–5 days before birth; we used the term “rodent” to represent all species from which we obtained experimental data; however, this does not imply that the models represent rodent ventricular cells in general. Table 1 lists the membrane currents that quantitatively change during embryonic development. The relative densities were as reported previously [10], except for the rapid component of the delayed rectifier K^+^ current (*I*_Kr_) and the background nonselective cation current (*I*_bNSC_). The relative density of *I*_Kr_ in the EE ventricular cell was originally set to 10.0 times that of the adult stage in our previous study [10], on the basis of the qualitative observation using the selective *I*_Kr_ blocker in EE rats [16, 17], and the models were constructed on the basis of the previous version of the Kyoto model [11]. In this study, we set the relative density of *I*_Kr_ in the EE stage to 2.0 times that of the adult stage because the amplitude of *I*_Kr_ conductance was increased by fourfold to reconstruct the AP duration prolongation through the pharmacological inhibition of *I*_Kr_ in the current version of the Kyoto model [15]. The relative density of *I*_Kr_ in the LE stage was also set to 2.0 times that of the adult stage on the basis of the qualitative observations using the selective *I*_Kr_ blocker in LE rats [18] and the *I*–*V* curve of the sum of *I*_Kr_ and *I*_Ks_ in LE guinea pigs [2]. *I*_bNSC_ was assumed to have a constant current density during development.

**Table 1:** Relative densities for ionic currents obtained from the literature

	EE	Reference	LE	Reference
*I*_Na_	0.07	[3]	1.00	[3]
*I*_CaL_	0.46	[22]	0.78	[2]
*I*_K1_	0.11	[36]	1.00	[2]
*I*_Kr_	2.0	[16, 17]	2.0	[2, 18]
*I*_KATP_	0.32	[3, 37]	0.88	[37, 38]
*I*_to_	0.01	[3]	0.27	[38]

The densities relative to that in the adult stage of the Na^+^ current (*I*_Na_), L-type Ca^2+^ current (*I*_CaL_), inward rectifier K^+^ current (*I*_K1_), ATP-sensitive K^+^ current (*I*_KATP_), and transient outward current (*I*_to_) for the early embryonic (EE) and late embryonic (LE) stages were estimated from the current–voltage (*I*–*V*) curves of cells in vitro. The *I*–*V* curve for *I*_Na_ was obtained from 11- to 13-day postcoitum (dpc), and 17- to 20-dpc mice [3]. For *I*_CaL_, the early embryonic *I*–*V* curve was obtained from 9.5-dpc mice [22]; the late embryonic *I*–*V* curve was obtained from 18-dpc mice [22] and fetal guinea pigs 1–7 days before birth [2]. For *I*_K1_, *I*–*V* curves were obtained for the 12-dpc rat [36] and the fetal guinea pig 1–7 days before birth [2]. The relative densities of *I*_KATP_ were obtained on the basis of the data for the 12-dpc and 18-dpc rats [37]. For *I*_to_, *I*–*V* curves were obtained for 11-dpc mice [3] and 1-day-old rats [38]

As listed in Table 2, the conversion factors for *I*_f_ (*P*_f,K_ and *P*_f,Na_) were set to those of *I*_f_ in sinoatrial node (SAN) cells [19] for the EE stage and to 0 pA/pF mM for the LE stage, based on the *I*–*V* curves for *I*_f_ in embryonic mice [6]. The conversion factors for *I*_st_ (*P*_st,K_ and *P*_st,Na_) were arbitrarily set to half of those in SAN cells for the EE stage and to 0 pA/pF mM for the LE stage because there was no evidence for the presence of *I*_st_ in embryonic rodents.

**Table 2:** Estimated conversion factors for the funny current (*I*_f_) and sustained inward current (*I*_st_)

	SAN (pA/pF mM)	Reference	EE (pA/pF mM)	Reference	LE (pA/pF mM)	Reference
*P*_f,Na_	0.01379	[19]	0.01379	[6]	0	[6]
*P*_f,K_	0.05635	[19]	0.05635	[6]	0	[6]
*P*_st,Na_	0.007375	[19]	0.003588	Arbitrarily set	0	Arbitrarily set
*P*_st,K_	0.0043125	[19]	0.002156	Arbitrarily set	0	Arbitrarily set

The conversion factors for *I*_f_ and *I*_st_ and the permeability coefficients for Na^+^ and K^+^ (*P*_f,Na_, *P*_f,K_, *P*_st,Na_, *P*_st__K_) for the EE and LE stages were arbitrarily set on the basis of the *P*_f,Na_ and *P*_f,K_ in the sinoatrial node (SAN) model [19] and the expression of the mRNA encoding the *I*_f_ channels in the 9.5-dpc and 18-dpc mice [6]. The *P*_st,Na_ and *P*_st,K_ in the EE model were assumed to be approximately half those in the SAN model [19]

SR development in ventricular cells of developing rodents is highly correlated with gestational age (Fig. 1): guinea pigs, which have a fairly long gestation period (67 days), have an almost fully developed SR at birth, whereas rabbits and rats have an immature SR that increases in size during postnatal development by 3- and 5-fold, respectively, and reaches adult size in a few weeks [20]. The relative amount of SR Ca^2+^ pump proteins in rabbit [21] and mouse [22] and that of the ryanodine receptor (RyR) channel in mouse [22] were adopted to be representative of SR Ca^2+^ kinetics for the rodent LE stage (Table 3). Similarly, we computed the average relative amount of the Na^+^/Ca^2+^ exchanger in rabbit [20] and mouse [22]. In addition, the Ca^2+^-induced activation rate for the RyR channel was multiplied by the average relative density values for SR Ca^2+^ kinetics to represent changes in the Ca^2+^-induced Ca^2+^ release (CICR) factor during embryonic development [4].

**Table 3:** Relative ratios of ion fluxes in exchanger, pump, and sarcoplasmic reticulum (SR) Ca^2+^ kinetics

	EE	Reference	LE	Reference
Na^+^/Ca^2+^ exchange	4.95	[22]	2.0	[20, 22]
SR Ca^2+^ pump	0.03	[22]	0.21	[21, 22]
RyR channel	0.05	[22]	0.40	[22]
SR Ca^2+^ transfer	0.04	Arbitrarily set	0.30	Arbitrarily set
SR Ca^2+^ leak	0.04	Arbitrarily set	0.30	Arbitrarily set
CICR factor	−3	Arbitrarily set	−60	Arbitrarily set

Developmental changes in Na^+^/Ca^2+^ exchange (*I*_NaCa_) have been reported as western blots of the NCX1 protein in rabbit [20] and mouse [22]. Based on findings implying that postnatal quantitative changes in the density of *I*_NaCa_ are in good agreement with changes in protein production levels [35], we assumed that the relative amounts of the proteins directly reflected the relative ratios of the ion fluxes of the *I*_NaCa_, SR Ca^2+^ pump, and RyR channel. Hence, the western blots of the SR Ca^2+^ pump proteins in rabbit [21] and mouse [22] and that of the RyR channel in mouse [22] were adopted for quantifying the relative amount of ion fluxes through the SR Ca^2+^ pump and RyR channel, respectively. Similarly, we computed the relative amount of ion flux through Na^+^/Ca^2+^ exchange on the basis of the relative amount of the Na^+^/Ca^2+^ exchanger protein in rabbit [20] and mouse [22]. The relative fluxes of Ca^2+^ transfer from the SR uptake site to the release site and Ca^2+^ leak from the SR were both set to 0.04 for the EE stage and 0.30 for the LE stage, based on the average of the relative amount of the RyR channel and that of the SR Ca^2+^ pump at the corresponding stages. Levels of the Ca^2+^-induced Ca^2+^ release (CICR) factor in the EE and LE stages were determined on the basis of the average relative density values for SR Ca^2+^ kinetics [4]

### Volumes of cell compartments

In the Kyoto model, 80 % of the total cell volume (*V*_t_, 16,000 μm^3^) is considered accessible for ion diffusion (*V*_i_, 12,800 μm^3^) [15]. The volumes of the cell compartments of the EE ventricular cell were computed as described previously [10]; the volumes of the SR uptake site (*V*_up_) and the SR release site (*V*_rel_) were set to 3.394 and 1.3576 μm^3^, respectively, based on the *V*_i_ in the EE ventricular cell model (*V*_i,EE_, 1697 μm^3^). In addition, the updated Kyoto model included the mitochondrion as a cell compartment in addition to the SR, and the mitochondrial volume (*V*_mit_) was set to 23 % of *V*_i_ [15]. Because the mitochondria are marginally developed in rodent EE ventricular cells, we arbitrary set the *V*_mit_ for the EE ventricular cells to 2.3 % of *V*_i,EE_ (39.031 μm^3^), which is 1/10 the ratio in the adult stage.

### Switching the relative densities of the ionic components

We selected the following nine components to be switched between the EE and LE stages: *I*_f_, *I*_st_, *I*_K1_, Na^+^ current (*I*_Na_), L-type Ca^2+^ current (*I*_CaL_), Na^+^/Ca^2+^ exchange current (*I*_NaCa_), transient outward current (*I*_to_), ATP-sensitive K^+^ current (*I*_KATP_), and a set of 4 electrical components of the SR. The electrical components of the SR—which included the permeability of Ca^2+^ release from SR to the dyadic space through the RyR channel (*I*_RyR_), Ca^2+^ leak from the SR (*I*_SR,leak_), the SR Ca^2+^ pump, and Ca^2+^ transfer from the SR uptake site to the release site (*I*_SR,transfer_)—were treated as a set of components in the SR because all 4 components are located in the SR and develop along with the development of the SR. The other ionic components in the model were assumed to have constant current densities during embryonic development.

We applied the exact same procedure to simulations with the TP human ventricular cell model [14], in which the following 7 components were switched between the EE and LE stages: *I*_Na_, *I*_f_, *I*_K1_, *I*_CaL_ *I*_st_, *I*_NaCa_, and SR-related components. Although the original TP model does not contain *I*_f_, we implemented a mathematical model for *I*_f_ [23]. Similarly, we implemented the same mathematical model for *I*_f_ [23] in the LRd model [12] and switched the *I*_Na_, *I*_f_, *I*_K1_, *I*_CaL_, and SR-related components between the EE and LE stages for further confirmation of our simulation with the Kyoto and the TP models.

### Model assumptions

We assumed that the 9 components in the Kyoto model switched relative densities directly from EE to LE values without intermediate levels, independently from the other components; therefore, we first tested 512 (2^9^) combinations of the model. We then classified the simulation results for the 512 combinations according to their electrical activities and also compared the simulated results in terms of contractile force. The Kyoto model adopted a 4-state contraction model [24] to simulate cardiac cell contraction; the authors of the Kyoto model also assumed that all transition steps from cross-bridge-formed states ([T*] and [TCa*]) to cross-bridge-released states ([T] and [TCa]) are ATP-dependent, because ATP binding to a myosin head disrupts the cross-bridge formation between myosin and actin [24, 25]. Although we adopted the value of half sarcomere length (hSL, μm)—computed using the model proposed by Negroni and Lascano [23]—as a quantitative parameter for cell contraction, this value is not completely quantitatively accurate because we did not consider the developmental changes in contractile proteins.

The Kyoto model contains a β1-adrenergic signaling cascade in which binding of isoproterenol (Iso) to the β1-adrenergic receptor activates protein kinase A (PKA) through activation of adenylate cyclase, and the activated PKA modulates *I*_CaL_, *I*_Ks_, phospholamban, SR Ca^2+^-ATPase (SERCA), and plasma membrane Ca^2+^-ATPase [15]. The application of iso-enhanced *I*_CaL_ density had a minimal effect on the EE ventricular myocytes but had strong effects on LE and adult ventricular myocytes, as observed in an experimental study [26]. Differences in β1-adrenergic modulation between EE and LE ventricular myocytes were not considered in this study because we focused on membrane excitation and contraction to present an overview of the functional landscape of developmental changes in embryonic ventricular cells.

### Computer simulation procedures

We switched the relative conductances of the 9 components between EE and LE values, and simulated the 512 combinations of the Kyoto model for 600 s with no external stimuli. For those combinations that showed no spontaneous activity for 600 s, we applied an external stimulation of −38 pA/pF, which is −8,000 pA divided by cell capacitance of the original Kyoto model (211.2 pF), at 2.5 Hz to determine whether the cells functioned as passive contracting cells. The exact same simulation procedures were applied to the TP and LRd models; we first simulated 128 combinations of the TP model and 32 combinations of the LRd model for 600 s, and provided additional 600-s simulations with external stimulation for those combinations without spontaneous activities. The external stimulation of −80 pA/pF was applied at a frequency of 2.5 Hz for the LRd model and that of −52 pA/pF at 1.0 Hz was applied for the TP model. The amplitudes of the external stimulation differed among the 3 models because we adopted the amplitudes that were used to evoke APs in the original models [12, 14, 15].

## Results

### Classification of the 512 combinations simulated with the Kyoto model

Of the 512 combinations simulated using the Kyoto model, 248 combinations were predicted to result in quiescent cells with no spontaneous activity; the external stimulus was applied at a frequency of 2.5 Hz for 600 s to pace the 248 combinations; however, 32 of them failed to fire APs. The evoked activities are illustrated as blue hysteresis loops in Suppl. Figs. 1 and 2; the loops begin at the upper left representing their RMP, and the membrane is then depolarized, overshooting the potential, and gradually repolarized to the RMP. The sarcomere length shortens during the repolarization phase; thus, the height of the hysteresis loop represents the force of contraction. In 64 combinations in which the relative densities of *I*_Na_, *I*_f_, and *I*_K1_ were set to LE values, an increase in the relative activities of *I*_CaL_ and SR-related components resulted in larger amplitudes of hSL and a decrease in *I*_NaCa_ resulted in smaller amplitudes of hSL. The exact same results were observed in the TP model; the amplitudes of Ca^2+^ transients were increased as the relative densities of *I*_CaL_ and SR-related components were increased to the LE values, and the amplitudes were decreased as the relative *I*_NaCa_ density was decreased to the LE values (Suppl. Fig. 2).

**Fig. 2:**
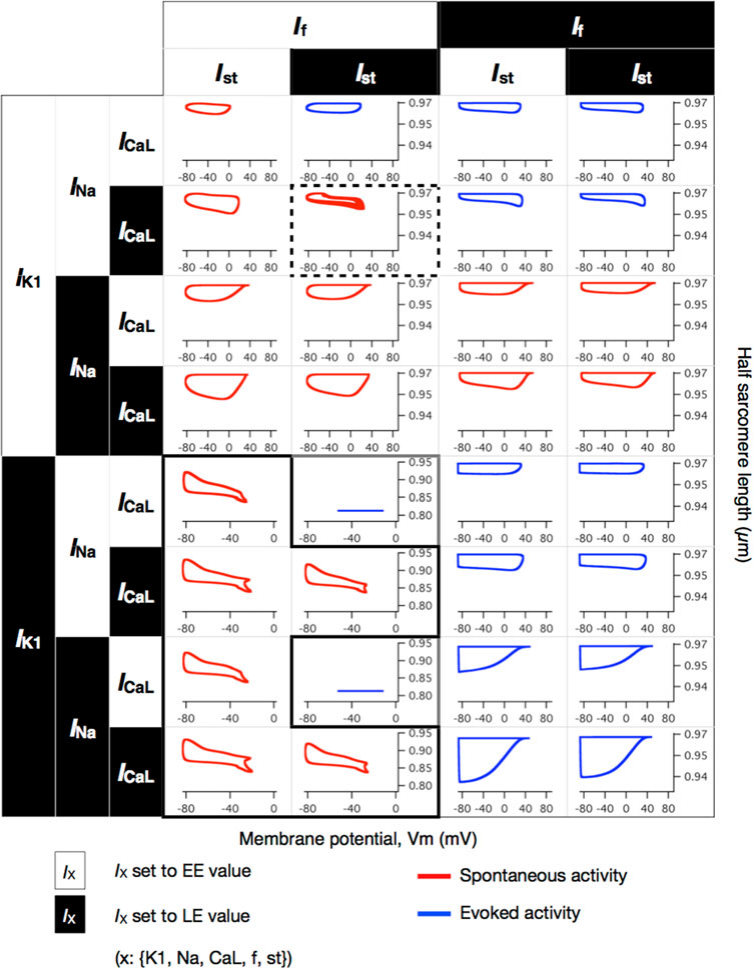
Simulated membrane potential and half sarcomere length (hSL) of 32 representative combinations in which the density values for 5 components were switched. The relative densities of *I*_Na_, *I*_f_, *I*_K1_, *I*_st_, and *I*_CaL_ were switched independently, yielding 32 combinations. The relative densities of the remaining 4 components were fixed to their EE values. Membrane potentials are presented on the *horizontal axis* and half sarcomere lengths are presented on the *vertical axis*. Models with membrane potential activities are presented as hysteresis loops, beginning at the *upper left* representing their maximum diastolic potential (MDP) and turning clockwise. Burst-like action potentials (APs) are highlighted with *dashed boxes*, shown in detail in Fig. 3a. Similarly, the combinations that were predicted to result in the spontaneous oscillation of the membrane potential with long AP duration are highlighted with the *solid black boxes*, shown in detail in Fig. 4c. The *red *hysteresis loops are illustrated on the basis of profiles from 600 to 605 s for the combinations with the regular spontaneous APs; the burst-like APs and the spontaneous oscillations with long AP duration were illustrated on the basis of the additional 600-s simulations. The *blue* hysteresis loops are illustrated on the basis of profiles from 599 to 600 s in the additional simulation with an external stimulus of 38 pA/pF at 2.5 Hz for 600 s

We observed 160 regular spontaneous APs, 96 spontaneous oscillations with long AP duration, and 8 burst-like APs. The BCL of the 160 regular spontaneous APs ranged from 306 to 884 ms. The spontaneous activities are illustrated as red hysteresis loops in Suppl. Figs. 1 and 2. Because the values of the *I*_KATP_, *I*_to_, *I*_NaCa_, and SR-related components did not significantly influence the simulation results in terms of spontaneous electrical activities, we focused on the remaining 5 pivotal currents—*I*_Na_, *I*_f_, *I*_K1_, *I*_st_, and *I*_CaL_—and 32 combinations (Fig. 2) as representatives of the 512 combinations.

The changes in *I*_f_ and *I*_K1_ in other models—the TP and LRd models—demonstrated similar results in terms of regular spontaneous APs (Suppl. Figs. 3 and 4); the shifts in relative densities of *I*_f_ and *I*_K1_ to LE values terminated the spontaneous APs, which were observed when the relative densities of both *I*_f_ and *I*_K1_ were set to the EE values. The APs were not evoked by the external stimulus, when the relative densities of *I*_K1_ and *I*_st_ were set to the LE values and those of *I*_f_ and *I*_CaL_ were set to the EE values in the Kyoto model (Fig. 2, solid light boxes); similar results were observed in both the TP and LRd models, when the relative density of *I*_K1_ was set to the LE value and that of *I*_f_ was set to the EE value, regardless of the remaining components (Suppl. Figs. 3 and 4). The spontaneous oscillations with long AP duration were observed only in the Kyoto model (Fig. 2, solid black box). The burst-like behaviors were observed in 10 combinations simulated using the TP model; in the LRd model, however, all 16 combinations for which the relative density of *I*_K1_ was set to the EE value showed regular spontaneous APs, and the burst-like behaviors were not observed.

### Burst-like membrane potentials in the Kyoto and TP models

**Fig. 3:**
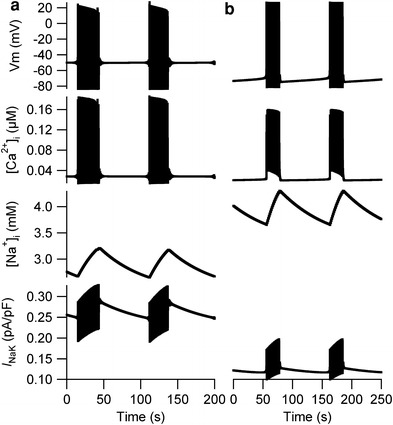
Burst-like membrane potentials in the Kyoto and Ten Tusscher–Panfilov (TP) models. **a** Burst-like activity was observed when the relative densities of *I*_st_ and *I*_CaL_ were set to the LE values and those of the other components were set to the EE values in the Kyoto model. **b** The burst-like activity was observed when the relative densities of *I*_f_ and *I*_NaCa_ were set to the LE values and those of *I*_K1_ and *I*_st_ were set to the EE values in the TP model

Simulations for eight combinations demonstrated burst-like APs when the relative densities of *I*_st_ and *I*_CaL_ were set to the LE values and those of *I*_Na_, *I*_f_, *I*_K1_, and *I*_NaCa_ were set to the EE values in the Kyoto model (Suppl. Fig. 1). In the representative combination in Fig. 3a, the duration of repetitive bursts was approximately 30 s and the amplitude of the membrane potential was approximately 100 mV. The interval between the bursts was approximately 70 s at −50 mV. Similar burst-like APs were also observed in simulations using the TP model (Fig. 3b), when the relative densities of *I*_f_ and *I*_NaCa_ were set to the LE values and those of *I*_K1_ and *I*_st_ were set to the EE values (Suppl. Fig. 3); the duration of repetitive bursts was approximately 20 s and the amplitude of the membrane potential was approximately 90 mV. The interval between the bursts was approximately 80 s at −70 mV.

The [Na^+^]_i_ was increased during repetitive bursts and decreased during the quiescent state between bursts in both the Kyoto and the TP models. In the Kyoto model, the [Na^+^]_i_ was 4.14 mM when the relative densities of all ionic components were set to EE values; the [Na^+^]_i_ was 3.2 mM when the burst was terminated and decreased to 2.7 mM during the quiescent state. In the TP model, however, the [Na^+^]_i_ was 4.01 mM when the burst was terminated and decreased to 3.65 mM, which are both higher than 1.56 mM—the [Na^+^]_i_ when all relative densities were set to EE values. In both models, however, the amplitudes of *I*_NaK_ increased during repetitive bursts and gradually decreased during the quiescent state between bursts.

### Increase in *I*_K1_ before the disappearance of *I*_f_ resulted in high intracellular Ca^2+^ concentrations in all 3 models

In the Kyoto model, increase in *I*_K1_ to the LE value before the disappearance of *I*_f_ resulted in either the spontaneous oscillation of the membrane potential with long APs or quiescent membrane potentials at approximately −50 mV. Of the 128 combinations in which *I*_K1_ was increased before the disappearance of *I*_f_, 32 combinations in which the relative density of *I*_CaL_ was set to the EE value and that of *I*_st_ was set to the LE value were predicted to result in quiescent membrane potentials at approximately −50 mV, and the APs were not evoked upon external stimulus application (Fig. 2, solid light boxes). Similarly, in the TP and LRd models, the APs were not evoked upon external stimulus application when *I*_K1_ was increased before the disappearance of *I*_f_ (Suppl. Figs. 3 and 4). The average RMPs were approximately −62.9 mV in the TP model and −69.1 mV in the LRd model.

**Fig. 4:**
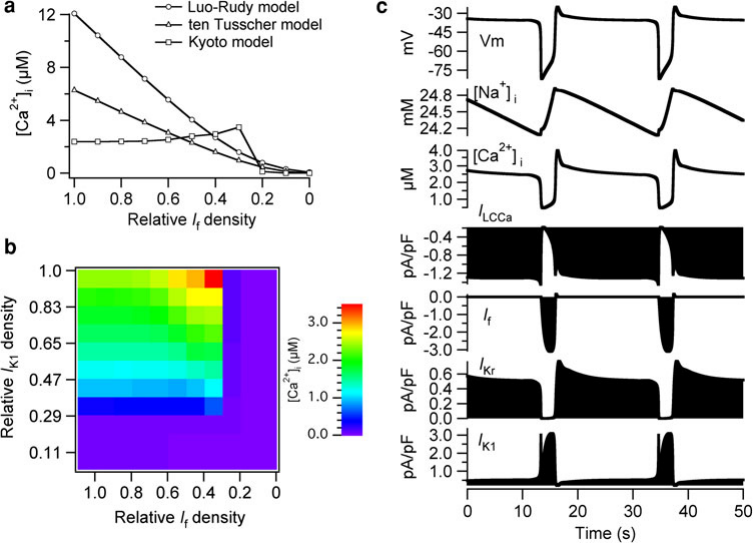
High intracellular Ca^2+^ concentrations were induced by an increase in *I*_K1_ before the disappearance of *I*_f_ in all 3 models. **a** The relative *I*_K1_ density was set to 1.0 (LE value) and the relative densities for all the other ionic components were set to the EE values. The relative *I*_f_ density was shifted by a 10 % interval from 1.0 to 0. For the Kyoto model, we calculated the average [Ca^2+^]_i_ during the additional 600-s simulation because [Ca^2+^]_i_ was not constant owing to the spontaneous oscillation of the membrane potential. **b** The relative densities of *I*_f_ and *I*_K1_ were shifted from EE to LE values independently by a 10 % interval, and the simulated [Ca^2+^]_i_ values are represented by *color* (range of *color bar*: 0.005–3.484 μM). **c** The spontaneous oscillation of membrane potential with long action potential duration was observed when the relative density of *I*_K1_ was set to the LE value and those of the other components were set to the EE values. The membrane potential oscillated between approximately −35 and −80 mV

In all 3 models, increase in *I*_K1_ to the LE value before the disappearance of *I*_f_ resulted in high [Ca^2+^]_i_ (Fig. 4a). The [Ca^2+^]_i_ was 12.1 μM in the LRd model and 6.27 μM in the TP model, and the decrease in the relative *I*_f_ densities decreased the [Ca^2+^]_i_ to 0.078 and 0.0065 μM, respectively. In the Kyoto model, we computed the average [Ca^2+^]_i_ during the additional 600-s simulations because [Ca^2+^]_i_ was not constant owing to the spontaneous oscillation of the membrane. The average of the simulated [Ca^2+^]_i_ was 2.38 μM when the relative *I*_f_ density was set to the EE value, and the average [Ca^2+^]_i_ increased to 3.48 μM as the relative *I*_f_ density decreased to 0.3; the [Ca^2+^]_i_ was then decreased to 0.0053 μM as the relative *I*_f_ density decreased from 0.3 to 0 (Fig. 4a). The contribution of the developmental changes in *I*_f_ and *I*_K1_ to [Ca^2+^]_i_ in the Kyoto model are further demonstrated in Fig. 4b, in which we shifted the relative densities of *I*_f_ and *I*_K1_ from EE to LE values independently by a 10 % increment. The average [Ca^2+^]_i_ was <0.05 μM when the relative *I*_K1_ density was set to <0.29 (30 % shift to LE), regardless of the relative *I*_f_ density; the [Ca^2+^]_i_ gradually increased from 0.35 to 2.38 μM as the relative density of *I*_K1_ was increased from 0.29 to 1.0.

In the Kyoto model, the increase in [Ca^2+^]_i_ resulted in the spontaneous oscillations of the membrane potential with long APs (Fig. 4c); the membrane potential spontaneously oscillated approximately every 20 s between −35 and −80 mV. The [Na^+^]_i_ oscillated between 24.2 and 24.9 mM, which is considerably higher than the original [Na^+^]_i_ (4.14 mM) in the original EE model; the [Na^+^]_i_ was also increased from 9.16 to 68.9 mM in the LRd model and from 1.56 to 60.9 mM in the TP model (data not shown). The [Ca^2+^]_i_ also oscillated at high concentrations, from 0.46 to 3.9 μM. The membrane was depolarized to −35 mV when *I*_f_ began to apply an inward current, followed by activation of the Ca^2+^-activated background cation current (*I*_LCCa_), which is activated when the intracellular Ca^2+^ concentration ([Ca^2+^]_i_) is high. The membrane potential was maintained at −35 mV for approximately 20 s when the amount of the outward K^+^ current, i.e., the sum of *I*_Kr_ and *I*_K1_, was approximately the same as that of *I*_LCCa_. The rapid increase in *I*_K1_ followed by deactivation of *I*_LCCa_ subsequently led to the repolarization of the membrane to −80 mV.

### Contribution of *I*_Na_ and *I*_f_ to the BCL of regular spontaneous APs

Of the 512 combinations simulated using the Kyoto model, 160 combinations were predicted to result in regular spontaneous APs. The BCL of the 160 regular spontaneous APs ranged from 306 to 884 ms; the maximum diastolic potential (MDP) ranged only from −85.0 to −80.9 mV, and the overshoot potential ranged from 1.6 to 54.3 mV. Of the 9 components that were shifted between EE and LE values, the developmental changes in *I*_Na_ and *I*_f_ made large contributions to the variation in the BCL and the overshoot potential in the Kyoto model; therefore, we further tested the contribution of the developmental changes in *I*_Na_ and *I*_f_ to the BCL and the overshoot potential.

**Fig. 5:**
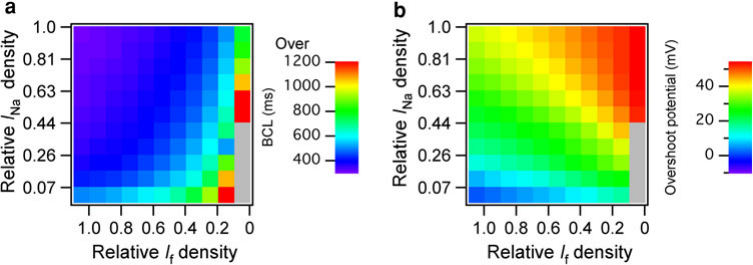
Contributions of *I*_Na_ and *I*_f_ to basic cycle length and overshoot potential of regular spontaneous action potential. The relative densities of *I*_f_ and *I*_K1_ were shifted from EE to LE values independently by a 10 % interval, and the simulated basic cycle lengths (**a**) and overshoot potential (**b**) were represented by *color*; range of color bar: (**a**) 300–1,200 ms and (**b**) 0–53.97 mV. The *gray boxes* represent the combinations with which spontaneous action potentials were terminated

As shown in Fig. 5a, the BCL of the regular spontaneous APs ranged from 314 to 1,550 ms in 121 combinations for which relative densities of *I*_Na_ and *I*_f_ were shifted independently by a 10 % increment from EE to LE values. The spontaneous APs ceased when the relative *I*_f_ density was set to 0 (LE value) and the relative *I*_Na_ density was set to <0.44 (40 % shift to LE), which are illustrated as gray boxes in Fig. 5. The increase in relative *I*_Na_ density from 0.07 to 1.0 shortened the BCL, and the decrease in relative *I*_f_ density from 1.0 to 0 prolonged the BCL; therefore, the longest BCL (1550 ms) was observed when the relative *I*_Na_ density was set to 0.07 (EE value) and the relative *I*_f_ density was set to 0.1 (90 % shift to LE). The overshoot potential, on the other hand, ranged from 2.1 to 53.7 mV in the 121 combinations, and both the increase in relative *I*_Na_ density and the decrease in relative *I*_f_ density increased the overshoot potential (Fig. 5b).

### Representative developmental changes in APs as *I*_Na_, *I*_f_, and *I*_K1_ were sequentially switched

**Fig. 6:**
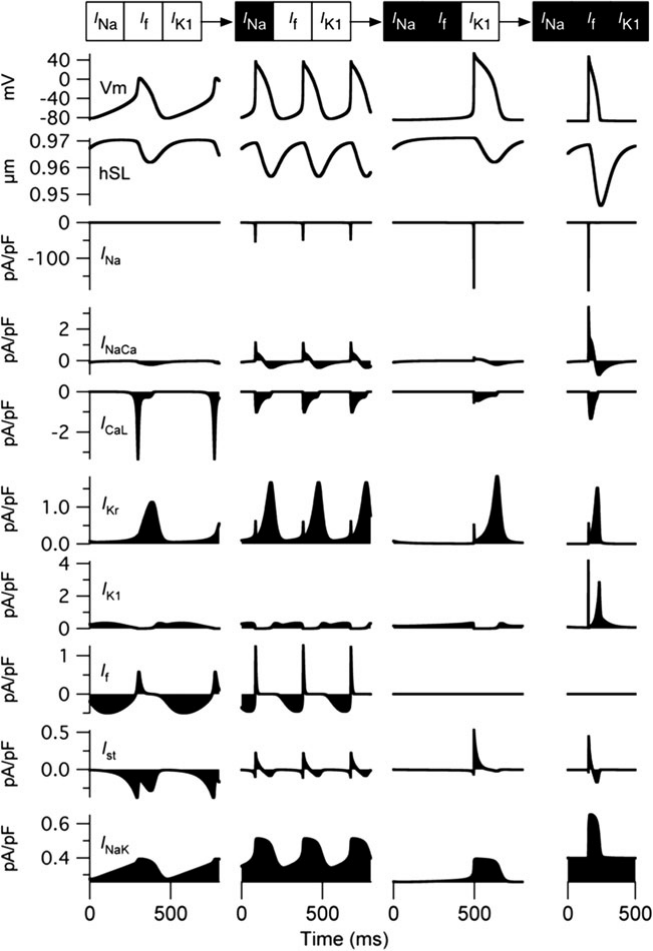
Simulated tracings of action potentials (APs), half sarcomere length (hSL), and accompanying ionic currents. The relative densities of *I*_Na_, *I*_f_, and *I*_K1_ were sequentially switched to the LE values from the EE model. An external stimulus (−38 pA/pF) was applied to the model in which the relative densities of *I*_Na_, *I*_f_, and *I*_K1_ were all set to the LE values

Figure 6 summarizes the changes in the APs and accompanying ionic currents as we sequentially switched *I*_Na_, *I*_f_, and *I*_K1_ from the EE model; spontaneous APs disappeared when *I*_Na_, *I*_f_, and *I*_K1_ were all switched to the LE values, although AP was inducible by external stimuli. The MDP gradually shifted to the negative direction and the overshoot potential became larger as *I*_Na_ increased, followed by the disappearance of *I*_f_ (Table 4). The BCL was originally 510 ms in the EE model, and the increase in *I*_Na_ shortened the BCL to 340 ms, which was the shortest among the 3 representative spontaneous APs.

**Table 4:** Characteristics of representative spontaneous action potentials

Combinations	MDP (mV)	Overshoot potential (mV)	BCL (ms)	DSD (ms)
EE model	−81.40	2.14	510	300
*I*_Na_ set to LE	−82.48	37.16	340	130
*I*_Na_ and *I*_f_ set to LE	−84.73	53.66	780	520

Values were determined from the last spontaneous AP of the 600-s simulation

MDP maximum diastolic potential, *BCL* basic cycle length, *DSD* diastolic slow depolarization

In the EE model, *I*_CaL_ was responsible for rapid depolarization of the membrane to overshoot the potential. As we sequentially switched *I*_Na_, *I*_f_, and *I*_K1_ to the LE values, the peak amplitude of *I*_CaL_ decreased from approximately −3.57 to −0.38 pA/pF and that of *I*_Na_ increased from 0 to −178.57 pA/pF, and *I*_Na_ became responsible for rapid depolarization rather than *I*_CaL_. Although we observed variations in the inward currents responsible for rapid depolarization, there were only slight differences in the outward currents (*I*_Kr_, *I*_K1_, and *I*_NaK_) responsible for membrane repolarization.

## Discussion

We predicted membrane excitation patterns from among computer simulations of 512 combinations with the Kyoto model (Suppl. Fig. 1) and confirmed the simulated results with 2 other models, the TP model (Suppl. Figs. 2 and 3) and the LRd model (Suppl. Fig. 4). In all 3 models, [Ca^2+^]_i_ was increased to nonphysiological levels when *I*_K1_ was increased to the LE value before the disappearance of *I*_f_.

### Burst-like membrane potentials in the Kyoto and TP models

Burst-like APs were observed in 8 combinations when the relative densities of *I*_st_ and *I*_CaL_ were set to the LE values and those of *I*_Na_, *I*_f_, *I*_K1_, and *I*_NaCa_ were set to the EE values (Suppl. Fig. 1); the burst-like APs disappeared as one of *I*_Na_, *I*_f_, *I*_K1_, and *I*_NaCa_ densities was shifted to LE value. In the TP model, on the other hand, burst-like APs were observed in 8 combinations in which the relative densities of *I*_f_ and *I*_NaCa_ were set to LE values and those of *I*_K1_ and *I*_st_ were set to EE values, and 2 combinations in which the relative densities of *I*_Na_, *I*_f_, *I*_CaL_, *I*_NaCa_, and *I*_st_ were set to LE values, and that of *I*_K1_ was set to the EE value (Suppl. Fig. 3). Although the combinations in which burst-like APs were observed were completely different between the Kyoto and the TP model, we observed similar dynamic changes in [Na^+^]_i_, which was increased during the repetitive bursts and decreased during the quiescent state between bursts (Fig. 3). During repetitive bursts, the amplitude of *I*_NaK_ was increased, which then decreased the amplitude of APs. As the bursts were terminated, *I*_NaK_ gradually decreased and contributed to the gradual increase in membrane potential during the quiescent state between bursts.

A similar pattern exhibiting burst-like APs (Fig. 3) has been reported in the pulmonary vein of rodents [27], and such APs in the pulmonary vein are known to cause atrial fibrillation [28, 29]. In our simulations, burst-like membrane potentials were observed when the relative densities of *I*_st_ and *I*_CaL_ were set to the LE values and those of *I*_Na_, *I*_f_, and *I*_K1_ were set to the EE values in the Kyoto model. *I*_st_ has been reported as ionic currents of Na^+^ and K^+^ and has been observed only in SAN cells [30]; however, there is no evidence for the presence of *I*_st_ in embryonic ventricular cells. Furthermore, we have no evidence for burst-like activities in ventricular cells at any stages of development. Although it may be interesting to note that such burst-like APs were predicted using both the Kyoto and TP models, we cannot draw notable conclusions from the simulated results because the combinations in which burst-like APs were observed were completely different between the Kyoto and TP models.

### *I*_f_ should disappear before the increase in *I*_K1_ to avoid high intracellular Na^+^ and Ca^2+^ concentrations

We observed abnormally high [Ca^2+^]_i_ values in all three models when *I*_K1_ was increased before the disappearance of *I*_f_; the simulated [Ca^2+^]_i_ was 12.1 μM in the LRd model, 6.27 μM in the TP model, and oscillated between 0.46 and 3.9 μM in the Kyoto model, when the relative *I*_K1_ density was set to LE and all the other densities were set to EE. In the TP model, the outward K^+^ current was increased as the relative *I*_K1_ density was increased from 0.11 to 1.0. Although the increase in outward K^+^ current polarized the membrane, the net inward current through *I*_f_ depolarized the membrane; therefore, the RMP was approximately −62.9 mV when the relative *I*_K1_ density was increased before the disappearance of *I*_f_. The increase in the inward Na^+^ current through *I*_f_ subsequently increased [Na^+^]_i_, which decreased the amount of Ca^2+^ excluded from the cytoplasm through *I*_NaCa_. Therefore, the high [Ca^2+^]_i_ in our simulation is mostly attributed to the decrease in *I*_NaCa_.

In the Kyoto model, the high [Ca^2+^]_i_ further led to the activation of *I*_LCCa_ (Fig. 4c), a current whose open probability increases at high [Ca^2+^]_i_ and contributes to transient inward current [31]. The activated *I*_LCCa_ contributed to the depolarization of the membrane to −35 mV; subsequent activation of *I*_K1_ resulted in repolarization of the membrane to −80 mV. Although abnormally high [Na^+^]_i_ and [Ca^2+^]_i_ were observed in all three models, the spontaneous oscillation with long AP duration between −35 and −80 mV was observed only in the Kyoto model; therefore, we should note that such spontaneous oscillations are unlikely to be observed in actual embryonic ventricular cells.

In addition to the abnormally high [Ca^2+^]_i_, we observed that the RMP was slightly positive to −80 mV when *I*_K1_ increased before the disappearance of *I*_f_; the average RMP of 8 combinations in the LRd model was −69.1 mV and that of 32 combinations in the TP model was −62.9 mV. Of the 128 combinations in which *I*_K1_ was increased before the disappearance of *I*_f_ in the Kyoto model, the membrane potentials did not oscillate but were quiescent at approximately −50 mV in 32 combinations for which the relative *I*_CaL_ density was set to the EE value and the relative *I*_st_ density was set to the LE value. None of the combinations could fire APs even after the application of a large external stimulus in all 3 models because the depolarized membrane caused voltage-dependent inactivation of *I*_Na_, whereas abnormally high [Ca^2+^]_i_ caused Ca^2+^-dependent inactivation of *I*_CaL_. Although there is no evidence to suggest that quiescent cells from 12-day fetal rat hearts failed to evoke APs upon the application of external stimulus, it is worth noting that the predicted RMPs were roughly consistent with the experimental observations obtained from 12-day fetal rat hearts; Nagashima et al. [5] obtained both cells with spontaneous APs and quiescent cells from 12-day fetal hearts wherein the quiescent cells exhibited an RMP of −48.4 ± 1.8 mV, which is more positive than the RMP of quiescent cells from 18-day fetal hearts (−80.9 ± 1.8 mV) [5].

### Relative densities of *I*_Na_ and *I*_f_ determine the BCLs and the overshoot potentials of the regular spontaneous APs

Of the 9 components shifted between EE and LE values, the developmental changes in *I*_Na_ and *I*_f_ had large contributions to the variation in the BCL and the overshoot potential in the Kyoto model; the BCLs of the 160 regular spontaneous APs ranged from 306 to 884 ms and the overshoot potential ranged from 1.6 to 54.3 mV. We further shifted the relative densities of *I*_Na_ and *I*_f_ independently by a 10 % increment from EE to LE values and showed that the increase in *I*_Na_ shortened the BCL, whereas the decrease in *I*_f_ prolonged the BCL; the BCLs of the regular spontaneous APs were prolonged up to 1,550 ms when the relative *I*_Na_ density was set to 0.07 (EE value) and the relative *I*_f_ density was set to 0.1 (90 % shift to LE).

EE ventricles have a large range of BCLs—337–542 ms in 9.5-dpc mice [6, 8, 9] and 273–2,500 ms in 12.5-dpc rats [5, 7]—and the beating rhythms of embryonic ventricular cells are irregular as reported in both 11.5-dpc rat ventricular cells [5] and 18.5-dpc mouse ventricular cells with spontaneous APs [6]. Although our simulations could not reproduce the irregular spontaneous APs reported in both mouse and rat embryonic ventricular cells, our predicted BCLs were all within the range of BCLs reported in experimental studies. We also showed that the wide range of the BCLs reported in vitro can be described by shifting the relative densities of *I*_Na_ and *I*_f_.

### *I*_Na_ becomes responsible for membrane depolarization as *I*_Na_, *I*_f_, and *I*_K1_ are sequentially switched from EE to LE levels

The simulated results imply that the increase in the relative *I*_K1_ density before the disappearance of *I*_f_ results in high [Ca^2+^]_i_ in all three models. In addition, we showed that the relative densities of both *I*_Na_ and *I*_f_ determine the BCL and overshoot potential of the regular spontaneous APs. On the basis of all the observations, we illustrated the representative changes in APs in which *I*_Na_ was increased before the disappearance of *I*_f_, followed by an increase in *I*_K1_ (Fig. 6). Following the sequence with representative models, we observed that *I*_Na_ took over the role of *I*_CaL_, which was originally the current responsible for the depolarization of the membrane in the EE model. This change in the dependence of depolarization from the Ca^2+^ current to the Na^+^ current is consistent with experimental observations in rodent ventricular myocytes [32] in which the MDP shifted to a negative direction, also consistent with our simulation (Table 4). The changes in BCL were roughly consistent with experimental observations on rat embryonic hearts; the BCL of the proximal ventricle in the 11.5-dpc embryonic rat heart was shorter than that of the ventricle in the 10.5-dpc rat; however, the BCL was prolonged again in the 12.5-dpc rat [7].

Our hypothesis that an increase in *I*_Na_ density and disappearance of *I*_f_ should be observed in the early stage of embryonic development is supported by experimental observations that the densities of *I*_Na_ and *I*_f_ change earlier than those of other components [3, 6], including *I*_CaL_, *I*_K1_, *I*_NaCa_, and SR-related components [2, 20]. We demonstrate here that switching all the components in the mathematical model enabled us to simulate all possible combinations and identify pivotal component switches to describe the reported characteristics of embryonic ventricular cells. Our simulation procedure, together with experimental observations in the literature, will likely be useful in identifying the sequential regulation of gene or protein expression during development, which is difficult to determine through experimental data alone.

### Limitations

Our study has several limitations. The densities of ionic components were obtained from various rodents, including rats, mice, rabbits, and guinea pigs (Tables 1, 2, 3), and implemented in the Kyoto and LRd models, both of which represent guinea pig ventricular cells. Therefore, simulations with the Kyoto and LRd models represent electrical activities of rodent ventricular cells in general. Although the TP model represents human ventricular cells, we implemented the densities listed in Tables 1, 2, and 3 in order to confirm our simulations with the Kyoto model; thus, the simulation with the TP model is not intended to represent developmental changes in human embryonic ventricular cells. In addition, changes in mRNA subtypes of the genes encoding the *I*_f_ and *I*_K1_ currents were not considered; the gene responsible for *I*_f_ is known to switch from *HCN4* to *HCN2* [6], whereas that for *I*_K1_ switches from *Kir 2.2* to *Kir 2.1* [5] during embryonic development. Although the length of the sarcomere was adopted as an index for the force of contraction, we did not consider developmental changes in contractile proteins; α- and β-myosin heavy chains (MHC), for example, are coexpressed and equally abundant in early embryonic ventricular cells, but α-MHC becomes predominant in adult ventricular cells [33]. Therefore, the simulated changes in the length of the sarcomere may not be quantitatively accurate.

## Conclusions

The relative densities of ionic components in mathematical models were switched independently between the EE and LE stages to identify pivotal components to describe reported characteristics of embryonic ventricular cells; all simulations were conducted using three models, the Kyoto, TP, and LRd models. In all three models, our simulations suggested that the tenfold increase in *I*_K1_ before the disappearance of *I*_f_ results in abnormally high [Ca^2+^]_i_. The developmental changes in relative densities of *I*_Na_ and *I*_f_ had large contributions to the wide range of BCL values in the regular spontaneous APs. Of the remaining six components in the Kyoto model, increases in *I*_CaL_ and SR-related components were involved in the enhancement of cell contraction.

## Electronic supplementary material

Below is the link to the electronic supplementary material.

**Supplemental Figure 1.** Simulated membrane potential and half sarcomere length (hSL) for the 512 combinationsThe relative densities the 9 components, Na^+^ current (*I*_Na_), funny current (*I*_f_), inward rectifier K^+^ current (*I*_K1_), sustained inward current (*I*_st_), L-type Ca^2+^ current (*I*_CaL_), Na^+^/Ca^2+^ exchange current (*I*_NaCa_), ATP-sensitive K^+^ current (*I*_KATP_), transient outward current (*I*_to_), and sarcoplasmic reticulum (SR)-related components were switched independently to yield 512 combinations. The membrane potentials and hSLs are represented along the horizontal and vertical axes, respectively; the ranges of the axes are indicated in graphs. An external stimulus (38 pA/pF) was applied to the combinations shown as* blue* hysteresis loops at a frequency of 2.5 Hz.* Black letters in white boxes* in the column and row headers indicate that the relative density of the current was set to the early embryonic (EE) value, and* white letters in black boxes* indicate that the relative density of the current was set to the late embryonic (LE) value. (PDF 5332 kb)

**Supplemental Figure 2.** Simulated dynamics of intracellular Ca^2+^ concentration in 128 combinations using the Ten Tusscher–Panfilov model. The relative densities of *I*_Na_, *I*_f_, *I*_K1_, *I*_CaL_, *I*_st_, *I*_NaCa_, and SR-related components were switched independently between the EE and LE values. The* red* traces are illustrated from the time the first maximum diastolic potential (MDP) appeared after 600-s simulations. An external stimulation (−52 pA/pF at 1.0 Hz) was applied to the combinations without spontaneous activities. The paced simulations were conducted for additional 600 s to produce APs, represented as* blue* traces, which are illustrated from 600 to 602 s after the additional 600-s paced simulations. (PDF 858 kb)

**Supplemental Figure 3.** Simulated membrane potentials for 128 combinations using the Ten Tusscher–Panfilov model. The relative densities of *I*_Na_, *I*_f_, *I*_K1_, *I*_CaL_, *I*_st_, *I*_NaCa_, and SR-related components were switched independently between the EE and LE values. The* red* traces are illustrated from the time the first maximum diastolic potential (MDP) appeared after 600-s simulations. An external stimulation (−52 pA/pF at 1.0 Hz) was applied to the combinations without spontaneous activities. The paced simulations were conducted for additional 600 s to produce APs, represented as* blue* traces, which are illustrated from 600 to 602 s after the additional 600-s paced simulations. (PDF 699 kb)

**Supplemental Figure 4.** Simulated membrane potentials for 32 combinations using the Luo–Rudy model. The relative densities of *I*_Na_, *I*_CaL_, *I*_f_, *I*_K1_, and SR-related components were switched independently between the EE and LE values. Spontaneous action potentials (APs), represented as* red* traces, were observed when the relative density of *I*_K1_ was set to the EE value; the* red* traces are illustrated 1 s from the time the first maximum diastolic potential (MDP) appeared after 600-s simulations. Spontaneous APs disappeared when the relative density of *I*_K1_ was set to the LE value, and external stimulus (−80 pA/pF at 2.5 Hz) was applied for additional 600 s to produce APs, represented as* blue* traces, which are illustrated from 600 to 600.8 s after the additional 600-s paced simulations. (PDF 471 kb)
