# Supplementary figures and images for: Contribution of quantitative changes in individual ionic current systems to the embryonic development of ventricular myocytes: a simulation study
Source: J Physiol Sci. 2013 Jun 13;63(5):355–67. doi: 10.1007/s12576-013-0271-x (PMC3751412; doi:10.1007/s12576-013-0271-x)

Supplemental Figure 1

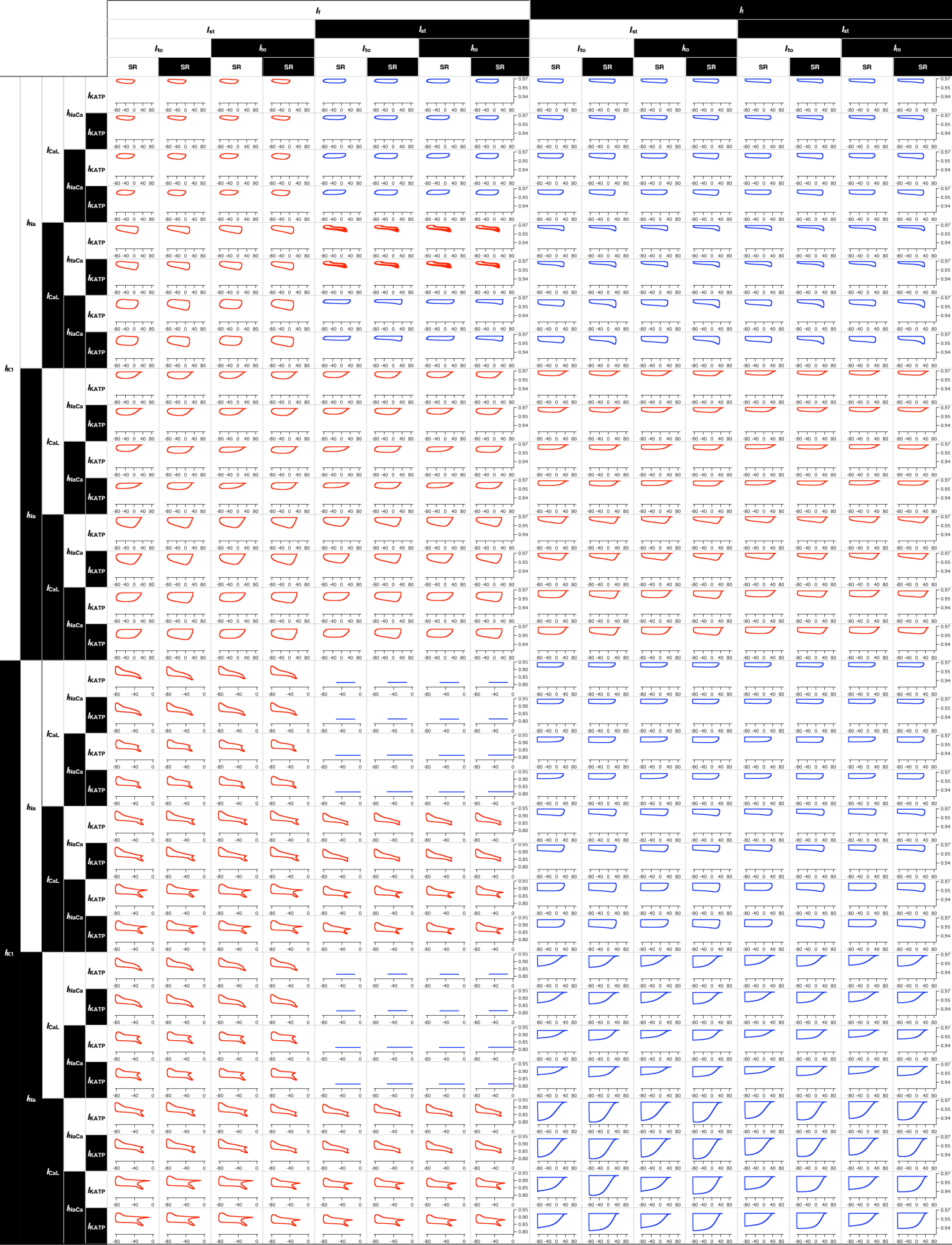

Supplement: Supplementary file 1 — Supplemental Figure 1. Simulated membrane potential and half sarcomere length (hSL) for the 512 combinationsThe relative densities the 9 components, Na+ current (I Na), funny current (I f), inward rectifier K+ current (I K1), sustained inward current (I st), L-type Ca2+ current (I CaL), Na+/Ca2+ exchange current (I NaCa), ATP-sensitive K+ current (I KATP), transient outward current (I to), and sarcoplasmic reticulum (SR)-related components were switched independently to yield 512 combinations. The membrane potentials and hSLs are represented along the horizontal and vertical axes, respectively; the ranges of the axes are indicated in graphs. An external stimulus (38 pA/pF) was applied to the combinations shown as blue hysteresis loops at a frequency of 2.5 Hz. Black letters in white boxes in the column and row headers indicate that the relative density of the current was set to the early embryonic (EE) value, and white letters in black boxes indicate that the relative density of the current was set to the late embryonic (LE) value. (PDF 5332 kb) [file 12576_2013_271_MOESM1_ESM.pdf]

Supplemental Figure 2

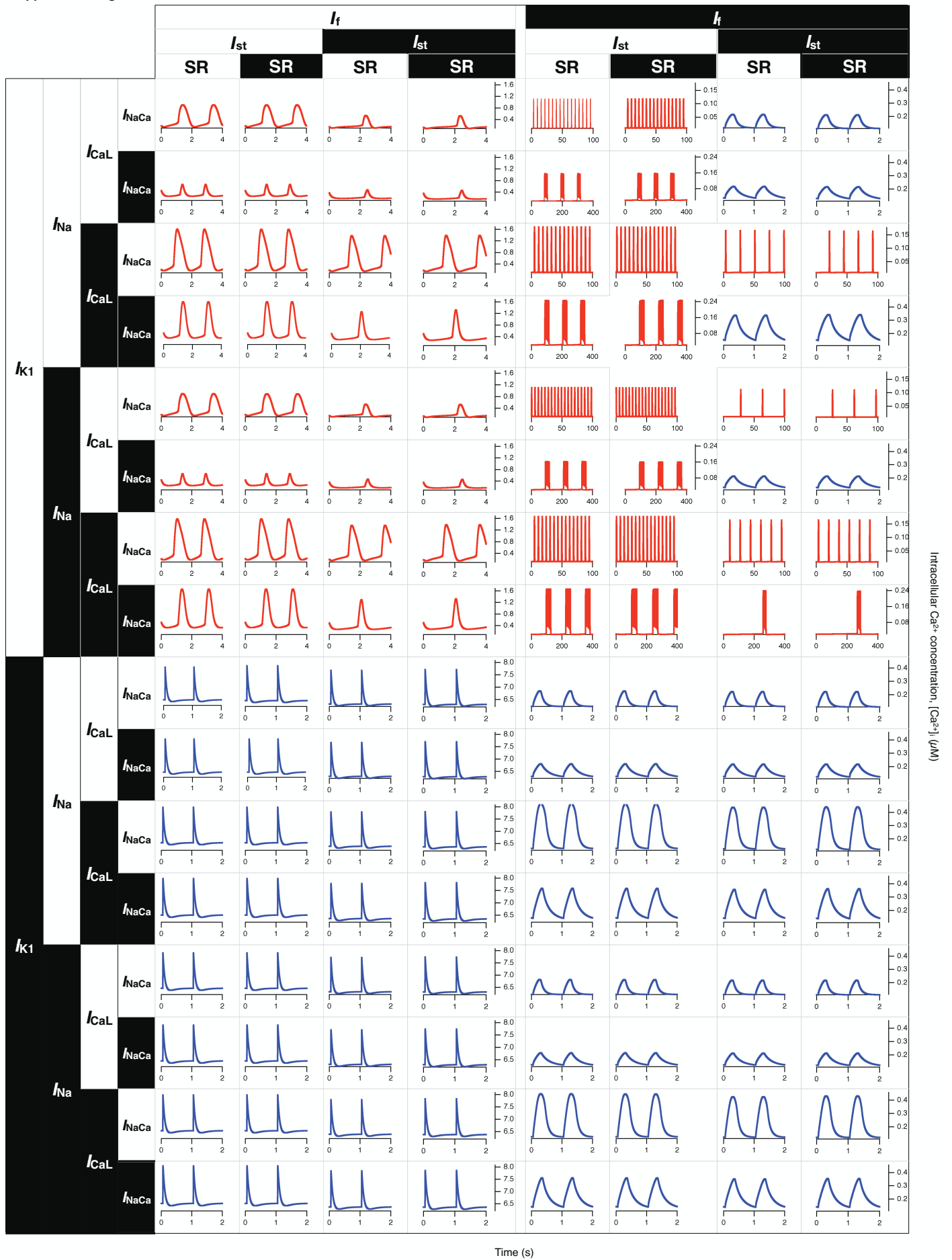

Supplement: Supplementary file 2 — Supplemental Figure 2. Simulated dynamics of intracellular Ca2+ concentration in 128 combinations using the Ten Tusscher–Panfilov model. The relative densities of I Na, I f, I K1, I CaL, I st, I NaCa, and SR-related components were switched independently between the EE and LE values. The red traces are illustrated from the time the first maximum diastolic potential (MDP) appeared after 600-s simulations. An external stimulation (−52 pA/pF at 1.0 Hz) was applied to the combinations without spontaneous activities. The paced simulations were conducted for additional 600 s to produce APs, represented as blue traces, which are illustrated from 600 to 602 s after the additional 600-s paced simulations. (PDF 858 kb) [file 12576_2013_271_MOESM2_ESM.pdf]

Supplemental Figure 3

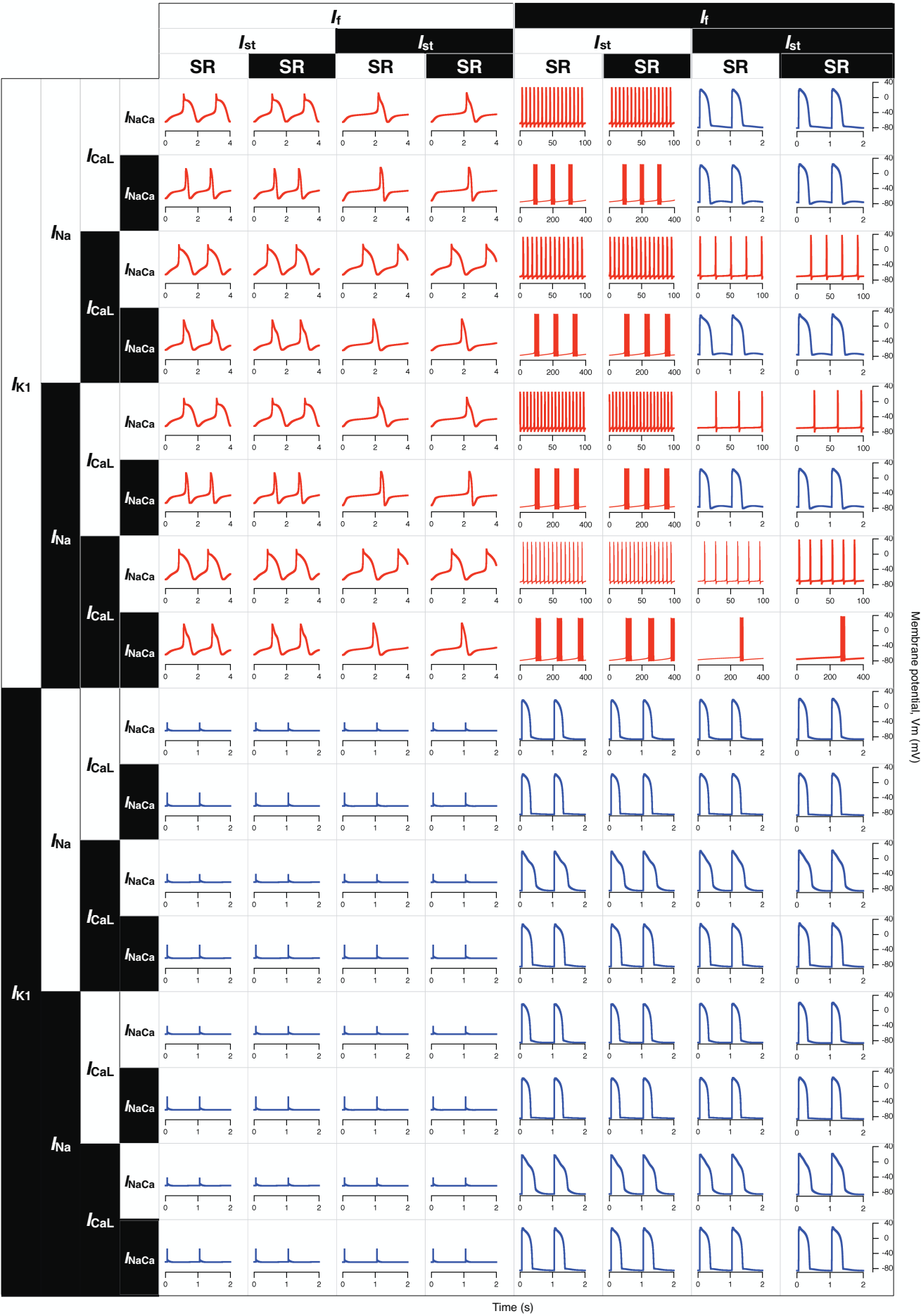

Supplement: Supplementary file 3 — Supplemental Figure 3. Simulated membrane potentials for 128 combinations using the Ten Tusscher–Panfilov model. The relative densities of I Na, I f, I K1, I CaL, I st, I NaCa, and SR-related components were switched independently between the EE and LE values. The red traces are illustrated from the time the first maximum diastolic potential (MDP) appeared after 600-s simulations. An external stimulation (−52 pA/pF at 1.0 Hz) was applied to the combinations without spontaneous activities. The paced simulations were conducted for additional 600 s to produce APs, represented as blue traces, which are illustrated from 600 to 602 s after the additional 600-s paced simulations. (PDF 699 kb) [file 12576_2013_271_MOESM3_ESM.pdf]

Supplemental Figure 4

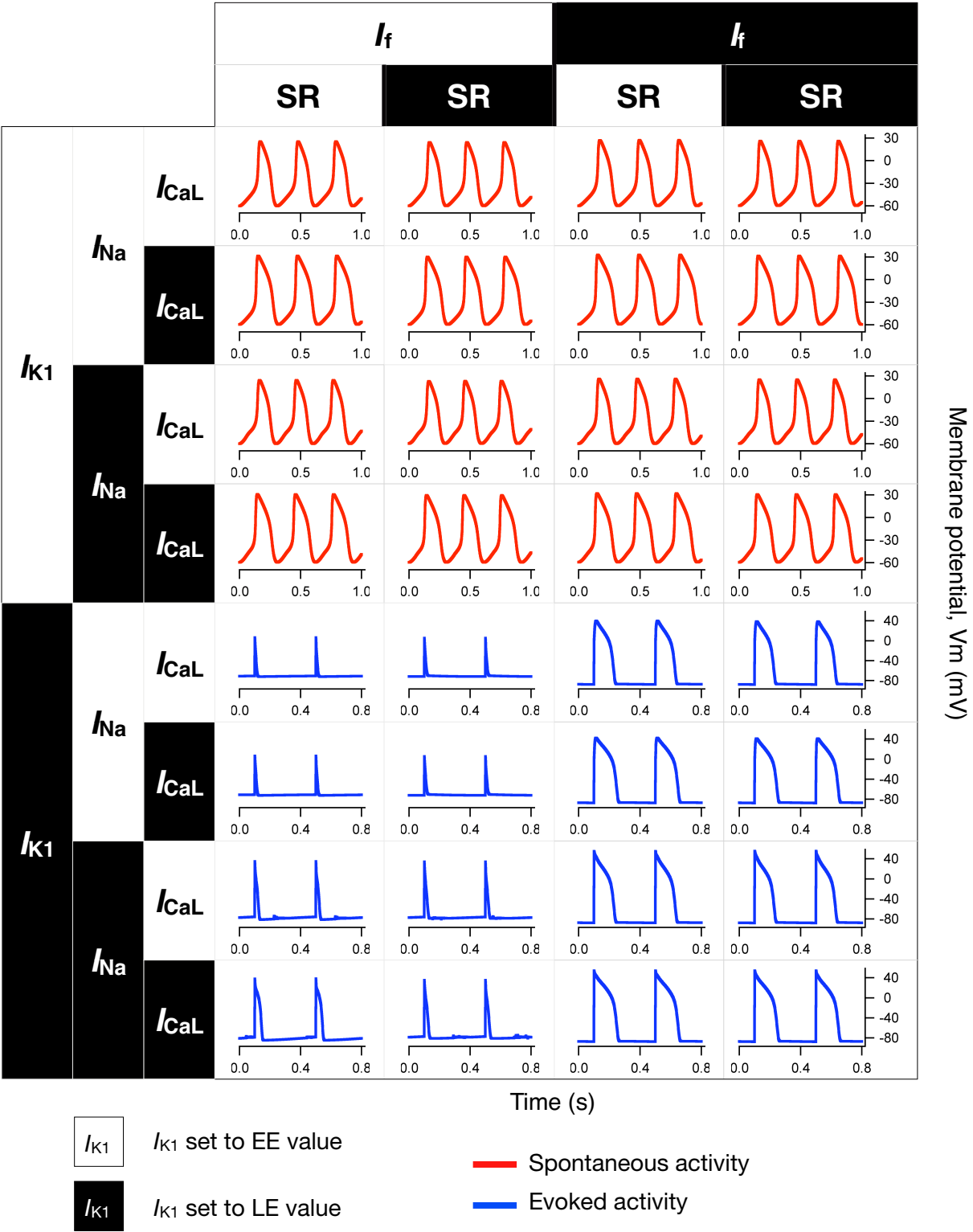

Supplement: Supplementary file 4 — Supplemental Figure 4. Simulated membrane potentials for 32 combinations using the Luo–Rudy model. The relative densities of I Na, I CaL, I f, I K1, and SR-related components were switched independently between the EE and LE values. Spontaneous action potentials (APs), represented as red traces, were observed when the relative density of I K1 was set to the EE value; the red traces are illustrated 1 s from the time the first maximum diastolic potential (MDP) appeared after 600-s simulations. Spontaneous APs disappeared when the relative density of I K1 was set to the LE value, and external stimulus (−80 pA/pF at 2.5 Hz) was applied for additional 600 s to produce APs, represented as blue traces, which are illustrated from 600 to 600.8 s after the additional 600-s paced simulations. (PDF 471 kb) [file 12576_2013_271_MOESM4_ESM.pdf]
